# Early presence of *Bythotrephes cederströmii* (Cladocera: Cercopagidae) in lake sediments in North America: evidence or artifact?

**DOI:** 10.1007/s10933-021-00213-w

**Published:** 2021-08-21

**Authors:** Nichole E. DeWeese, Elizabeth J. Favot, Donn K. Branstrator, Euan D. Reavie, John P. Smol, Daniel R. Engstrom, Heidi M. Rantala, Shawn P. Schottler, Andrew M. Paterson

**Affiliations:** 1 Department of Biology, University of Minnesota Duluth, 1035 Kirby Drive, Duluth, MN 55812 USA; 2 Paleoecological Environmental Assessment and Research Lab (PEARL), Department of Biology, Queen’s University, Kingston, ON K7L 3N6 Canada; 3 Natural Resources Research Institute, University of Minnesota Duluth, 5013 Miller Trunk Highway, Duluth, MN 55811 USA; 4 St. Croix Watershed Research Station, Science Museum of Minnesota, 16910 152nd St. N., Marine on St. Croix, MN 55047 USA; 5 Minnesota Department of Natural Resources, 5351 North Shore Dr, Duluth, MN 55804 USA; 6 Dorset Environmental Science Centre, Ontario Ministry of the Environment, Conservation and Parks, Dorset, ON P0A 1E0 Canada

**Keywords:** Invasive species, Invasion biology, *Bythotrephes cederströmii*, Paleolimnology, Spiny water flea

## Abstract

The spiny water flea (*Bythotrephes cederströmii*), a freshwater crustacean considered to be the world’s best-studied invasive zooplankter, was first recorded in North America in the Laurentian Great Lakes during the 1980s. Its arrival is widely considered to be the result of ocean-going cargo ships that translocated contaminated ballast water from Eurasia to the Great Lakes during the 1970–1980s. The subsequent first discovery of the species in inland lakes is consistent with the hypothesis that propagules dispersed initially from established Great Lakes populations. Here we present evidence of exoskeletal remains, including mandibles, tail spines, and resting eggs, in ^210^Pb-dated lake sediment cores, which suggests that *B. cederströmii* was already resident in four inland North American lakes (two in Minnesota, USA; two in Ontario, Canada) by at least the early 1900s. Densities of exoskeletal remains were low and relatively steady from first appearance until about 1990, after which time they increased in all cores. The earliest evidence that we found was a mandible at 33-cm depth (pre-1650) in the sediments of Three Mile Lake, Ontario, Canada. These unexpected findings challenge the current paradigm of *B. cederströmii* invasion, renew uncertainty about the timing and sequence of its colonization of North American lakes, and potentially question our ability to detect invasive species with traditional sampling methods. We attempted to eliminate errors in the dated stratigraphies of the exoskeletal remains that might have been introduced either methodologically (e.g., core-wall smearing) or naturally (e.g., bioturbation). Nonetheless, given the very low numbers of subfossils encountered, questions remain about the possible artifactual nature of our observations and therefore we regard our results as ‘preliminary findings’ at this time.

## Introduction

Nonindigenous invasive species are a leading threat to the preservation of Earth’s biodiversity and the structure and function of its ecosystems (Vitousek et al. 1997). In the Laurentian Great Lakes alone, more than 180 nonindigenous microbe, protist, plant, and animal species are believed to have successfully invaded since record-keeping began in the early 1800s (Mills et al. 1993; Ricciardi 2006; Sturtevant et al. 2019). Although early-detection tools are available for nonindigenous species, taxa that are in low abundance or have limited spatial distribution can escape discovery (Hoffman et al. 2011; Bailey et al. 2020), complicating our ability to track when and where their ranges expand. Changes in climate patterns and food web alterations can trigger populations of invasive species to expand (Spear et al. 2021).

Paleolimnology offers a compelling approach for studying invasive species, especially the events and processes that occurred prior to the beginning of records of direct observation and measurement (Smol 2019; Paterson et al. 2020). Past studies of colonization and extirpation of nonindigenous zooplankton have benefitted from paleolimnology (Hall and Yan 1997; Hairston et al. 1999; Kerfoot et al. 2004; Mergeay et al. 2007; Suchy and Hann 2007; Burillo et al. 2019) because zooplankton, particularly cladocerans, often leave behind rich exoskeletal evidence (Jeppesen et al. 2001) and genetic information (Burge et al. 2018). However, the preservation level of some species can vary in sediment (Leppänen and Weckström 2016).

*Bythotrephes cederströmii* is considered a nonindigenous zooplankton in North America that is native to Europe and Asia. It was first reported in plankton-net samples in North America in the 1980s (Johannsson et al. 1991; Yan et al. 2011) and since then has attracted the attention of a large spectrum of lake scientists and managers (Yan et al. 2011). Its impacts on aquatic food webs extend from top-down to bottom-up effects. *B. cederströmii* predation depletes native zooplankton species populations, which can cause algal populations to increase due to a reduction in grazing pressure. At the same time, reduction in native zooplankton species populations removes food sources for planktivorous fish and has been shown to reduce growth rates and populations of different fish species (Azan et al. 2015; Walsh et al. 2016a; Staples et al. 2017; Hansen et al. 2020).

Like other cladocerans, *B. cederströmii* are typically short-lived (days to weeks) and generally parthenogenetic. They reproduce clonally except during autumn when they birth males, then mate with them to produce resting eggs that serve to overwinter the population (Branstrator 2005). Individuals attain about 1 cm total length. Exoskeletal remains of their mandibles, tail spines, and resting eggs are diagnostic and have been recovered from the sediments of European and North American lakes and used to reconstruct historical population size (Nilssen and Sandoy 1990; Hall and Yan 1997; Milan et al. 2017). In Harp Lake, Ontario, Hall and Yan (1997) found a statistically indistinguishable difference between the annual population growth of *B. cederströmii* based on neo- and paleo-records. However, in Lago Maggiore, Italy, where *Bythotrephes longimanus* is present, researchers regularly detect the species in the water column, but have not recovered a single exoskeletal remain from sediment cores (Manca et al. 2007; Nevalainen et al. 2018), suggesting that gaps exist in our understanding of the relationship between the neo- and paleo-records for *Bythotrephes*.

**Fig. 1:**
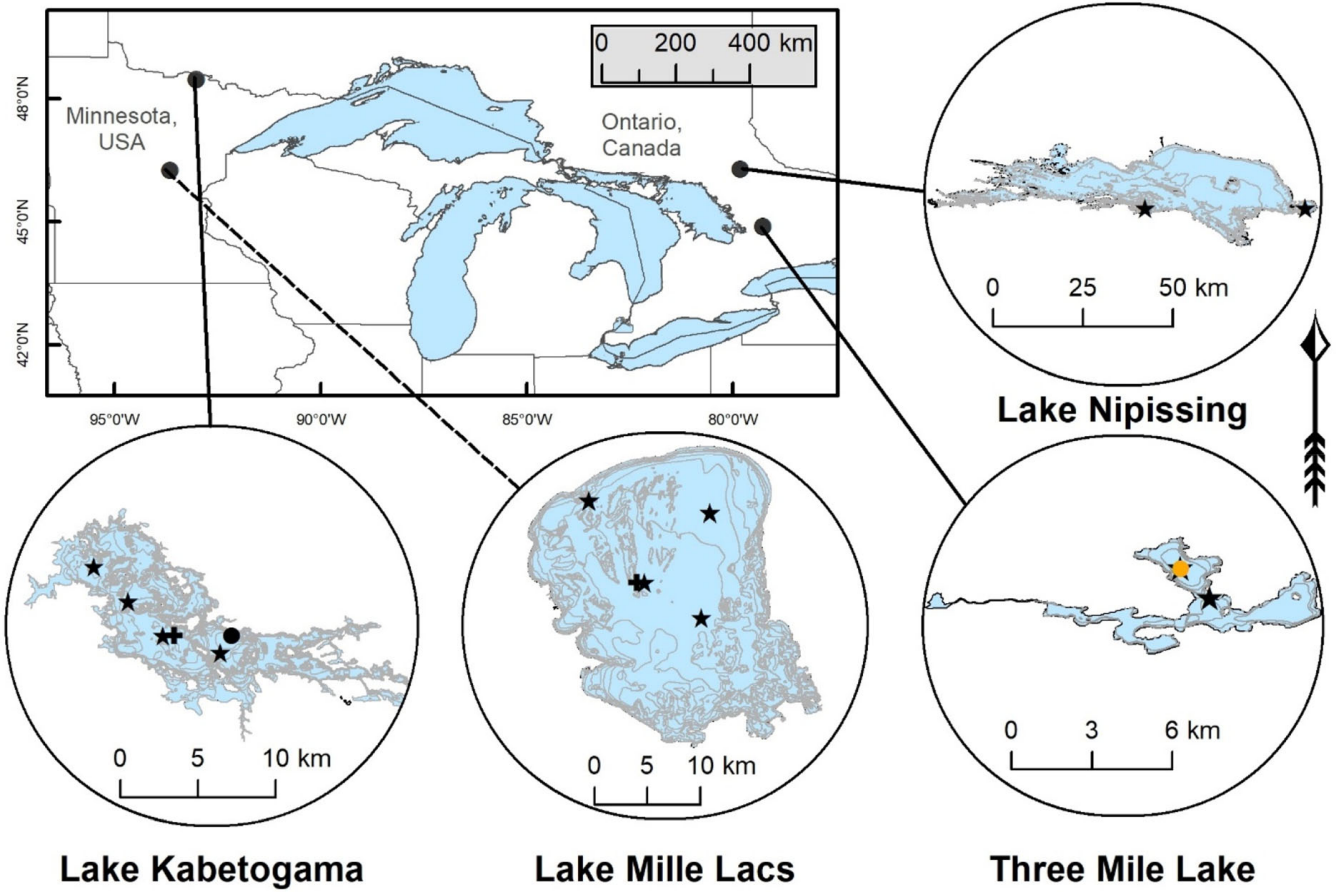
Map of lake and core locations. Coring sites are marked with a black star. Historical core locations in Lake Kabetogama are marked with a black circle. The historical core location in Three Mile Lake is marked with a yellow circle. Secondary cores for ^7^Be analyses in Lake Kabetogama and Lake Mille Lacs are marked with a black cross

We used paleolimnology to reconstruct early occurrences and patterns of colonization of *B. cederströmii* in four inland North American lakes including two in the USA and two in Canada (Fig. 1). Our analyses of exoskeletal remains indicate *B. cederströmii* appeared in North America decades prior to the accepted timeline of invasion on the continent (Bur et al. 1986; Sprules et al. 1990; Johannsson et al. 1991). Given the implications of our results, and the fact that we are dealing with very few specimens, we discuss the alternative possibility that they are the product of methodological or natural factors that led to errors in the dated stratigraphies of exoskeletal remains. Our field and laboratory methods were carried out by independent research teams working on Minnesota and Ontario lakes.

## Materials and methods

### Minnesota lakes

Lake Kabetogama (z_max_ = 24 m, surface area = 97 km^2^) and Lake Mille Lacs (z_max_ = 13 m, surface area = 519 km^2^) are located in Minnesota (USA) (Fig. 1). Zooplankton monitoring by net tow has occurred periodically in Lake Kabetogama since 1978 (Kallemeyn et al. 2003; Kerfoot et al. 2016) and in Lake Mille Lacs since 2006 (MNDNR, personal communication). *B. cederströmii* was first recorded in net-tow collections from the water column of Lake Kabetogama in 2007 and Lake Mille Lacs in 2009 (MNDNR 2018).

Sediment cores for dating and *B. cederströmii* recovery were collected from four sites in each lake (Table 1). Sites were chosen to maximize spatial coverage across the offshore and deeper regions of each lake. Sediment cores were collected from the ice surface in February and March of 2017 and 2018 using a piston corer outfitted with a 6.5-cm inside diameter, 1-m-long polycarbonate tube. Immediately after collection, each core was sealed with Zorbitrol at the sediment–water interface to stabilize the uppermost deposits of sediment (Tomkins et al. 2008). Cores were between 54 and 98 cm in length and all cores were transported to the University of Minnesota Duluth for extrusion and analysis.

**Table 1:** Coring site name, location, water column depth (m), down-core depth (cm) of the intervals corresponding to the year 2000, 1980, or 1930 (as estimated by ^210^Pb). Also shown are gravimetric results (% water and organic matter [OM] content) for the eight primary cores, designated as those searched for exoskeletal remains from Lake Kabetogama and Lake Mille Lacs. The loss-on-ignition results are given as mean ± 1 standard deviation for the uppermost 25 cm. Location is also reported for the secondary cores from Lake Kabetogama and Lake Mille Lacs, designated as those used for ^7^Be analysis, and for the historical cores searched for exoskeletal remains

Lake and core	Location			Down-core depth			Gravimetric measures	
	Lat	Long	Depth	2000	1980	1930	Water	OM
*Primary cores*								
Kabetogama 1	48.50	− 93.07	5.7	12	18	27	89.2 ± 3.7	29.7 ± 7.7
Kabetogama 2	48.48	− 93.04	8.1	11	16	27	90.7 ± 2.2	25.0 ± 2.8
Kabetogama 3	48.46	− 93.01	9.3	7	13	23	90.3 ± 1.6	22.9 ± 2.3
Kabetogama 4	48.45	− 92.96	15.1	9	17	30	90.1 ± 0.9	22.0 ± 2.2
Mille Lacs 1	46.32	− 93.75	8.9	1	2	5	93.2 ± 0.5	45.2 ± 1.8
Mille Lacs 2	46.25	− 93.68	10.1	3	5	9	92.7 ± 0.4	38.1 ± 2.1
Mille Lacs 3	46.22	− 93.61	10.9	5	9	18	93.5 ± 0.9	36.6 ± 3.3
Mille Lacs 4	46.31	− 93.60	9.4	3	5	10	92.4 ± 0.8	34.8 ± 2.9
Nipissing 1	46.21	− 79.39	10.0	2	4	8	–	–
Nipissing 2	46.21	− 79.79	36.6	8	14	24	–	–
Three Mile 1	45.19	− 79.47	3.7	3	6	10	–	–
Three Mile 2	45.18	− 79.46	12.0	4	7	11	–	–
*Secondary cores*								
Kabetogama	48.46	− 93.00	10.1	–	–	–	–	–
Mille Lacs	46.25	− 93.69	11.3	–	–	–	–	–
*Historical cores*								
Kabetogama H1	48.46	− 92.95	17.1	–	–	–	–	–
Kabetogama H2	48.46	− 92.95	16.7	–	–	–	–	–
Three Mile H1	45.19	− 79.47	11.5	–	–	–	–	–

Sediments were extruded in the laboratory using an incremental core-extruding apparatus. For the first 20 cm of sediment measured from the sediment–water interface, sediments were extruded in 0.5-cm increments and then in 1-cm increments for the remainder of the core. As the sediment was extruded, the outer 0.5-cm edge was trimmed using a slightly narrower-diameter plastic ring and discarded to reduce cross contamination among intervals caused by smearing along the inner edge of the core tube during collection and extrusion. Tools were cleaned between processing each increment. All sediment intervals were sealed in individual Whirl–pak® bags and stored at 5 °C until further analysis.

Sediment intervals were analyzed for water and organic matter gravimetrically at the University of Minnesota Duluth (Dean 1974) and dated with ^210^Pb at the St. Croix Watershed Research Station of the Science Museum of Minnesota. Briefly, water content was determined by measuring mass loss on heating sediment samples to 100 °C for 24 h. Dry samples were then heated to 550 °C for 2 h and re-weighed to determine organic matter content. Lead-210 was measured from freeze-dried samples at 17–23 depth intervals in each core through its grand-daughter product ^210^Po, with ^209^Po added to each sample (0.3–1.0 g) as an internal yield tracer. The polonium isotopes were distilled at 550 °C onto wetted quartz wool, refluxed in a 0.5 M HCl solution, and plated directly onto silver planchets (Eakins and Morrison 1978). Activity was measured for 1–12 days on an Ortec alpha spectroscopy system. Unsupported ^210^Pb was calculated by subtracting supported activity from the total activity measured at each level; supported ^210^Pb was estimated from the asymptotic activity at depth (the mean of the lowermost samples in a core). Dates and sedimentation rates were determined according to the CRS (constant rate of supply) model (Appleby 2001) with errors calculated by first-order propagation of counting uncertainty.

**Fig. 2:**
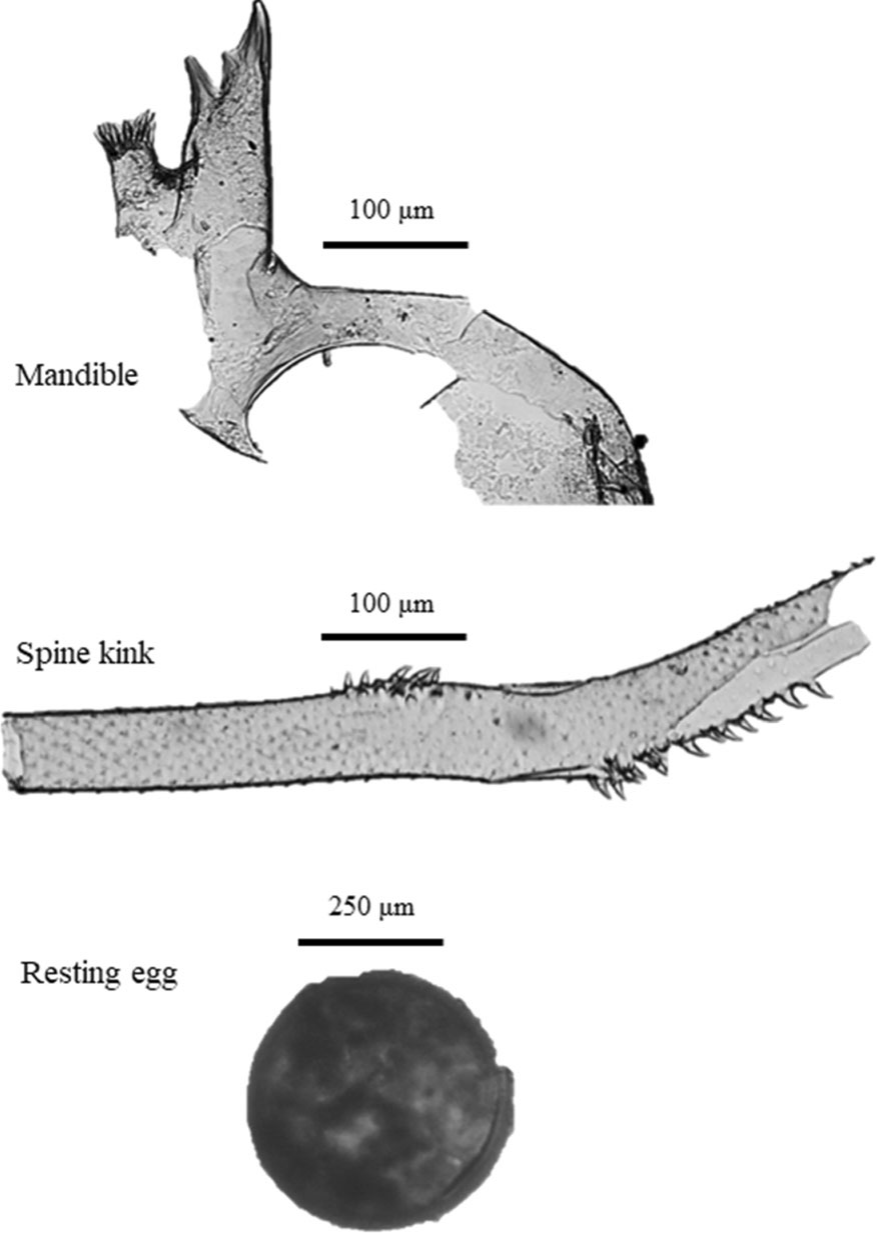
Examples of a *B. cederströmii* mandible, spine kink, and resting egg recovered from lake sediments

After subsamples were removed for loss-on-ignition measurements and ^210^Pb dating, remaining sediments were searched for *B. cederströmii* remains. Before searching, sediments were prepared by dilution in a mix of distilled water and Eosin Y (Fisher Scientific) stain for at least 4 h. Sediment was then filtered (210-µm Nitex mesh) to remove fine-grained material, which helped reduce search times. The filter was rinsed and examined under a microscope after each sample to ensure all subfossils were removed before the next sample was filtered. The dyed and filtered sediment solutions were searched under dissecting microscopes at 20x magnification. A dissecting microscope was used instead of a compound microscope to reduce search times, since *B. cederströmii* subfossils are large enough to be detected using a dissecting microscope. Searches on each sample were usually performed by two people as follows. A small amount of the sediment solution was poured onto a gridded Petri dish and the surface tension was broken with a few drops of soapy water. The dish was searched systematically across the grids and remains were removed and tallied. Once the dish was completely searched, the dish was gently agitated to move the sediment solution into a new position and searched again. Each dish was searched three times in this fashion by a single person, and then set aside to be searched an additional fourth time by a second person as a measure of quality control. If a second person was not available, the dish was searched once more by the original searcher. *B. cederströmii* remains included mandibles, complete tail spines, fragments of broken tail spines, and resting eggs (Fig. 2). Each tail spine contains a single kinked portion (Fig. 2) which we used here as the empirical estimate for numbers of tail spines recovered. Because the tail spine does not shed during body molting and instar progression, each kink represents a single dead individual.

Additional sediment cores were collected on both lakes to estimate depths of sediment mixing in the uppermost layers using ^7^Be. Previously collected cores could not be used for ^7^Be analysis because of the very short half-life (53 days) of this cosmogenic radionuclide. Detection of ^7^Be at depth (e.g., a few cm below the sediment surface) can be used to determine the extent of sediment mixing (Krishnaswami et al. 1980; Edlund et al. 2017). For this exercise, one sediment core from a single site in each lake was collected in open water from a boat in September 2019 (Table 1), using the same coring device as described above. At Lake Kabetogama, the core was capped, secured vertically, and returned to shore where it was immediately extruded. At Lake Mille Lacs, the water head space of the core was stabilized with Zorbitrol, and the entire core was returned to the University of Minnesota Duluth for extrusion. Cores were extruded in 1-cm increments, trimming the outer 0.5-cm edge with a plastic ring as described above. Fresh material was shipped immediately to the St. Croix Watershed Research Station, Minnesota, where ^7^Be was measured by gamma spectroscopy. ^7^Be was analyzed in each core at contiguous depth intervals from the top downward until several intervals without detectable ^7^Be were reached. Isotopic activities were measured at 477.56 kev for 7–30 × 10^4^ s using an EG&G Ortec high-resolution germanium well detector and multichannel analyzer. All samples were analyzed within 12 weeks of collection, and activities were decay-corrected to the date of coring.

Finally, for Lake Kabetogama we searched sediments from cores collected in 2001 and 2005, prior to the year of first detection of *B. cederströmii* in plankton net tows in 2007. The methods of collection for those cores are reported in Engstrom and Balogh (2005) and Edlund et al. (2014), and the collection locations are reported in Table 1 under Kabetogama H1 and Kabetogama H2. The historical core material had been freeze-dried prior to storage. We prepared it by soaking overnight in water, a small amount of dish soap, and Eosin Y. The hydrated sediment was filtered and searched as described above.

### Ontario lakes

Lake Nipissing (z_max_ = 52 m, surface area = 822 km^2^) and Three Mile Lake (z_max_ = 12 m, surface area = 8.8 km^2^) are located in central Ontario (Canada) (Fig. 1). *B. cederströmii* was first recorded in net-tow collections from the water column of Lake Nipissing in 1998 and Three Mile Lake in 2001 (MacIsaac et al. 2004).

Sediment cores for dating and *B. cederströmii* recovery were retrieved from two sites in each lake (Table 1) in June-July 2017 using a Glew (1989) gravity corer and sectioned on shore using a Glew (1988) extruder. The cores (29–38 cm in length) were sectioned at 0.5-cm resolution throughout, and tools were cleaned between processing each increment. Sediment slices were placed into Whirl–pak® bags and kept in coolers until returned to the cold room (~ 4 °C) at the Paleoecological Environmental Assessment and Research Laboratory (PEARL) at Queen’s University, Kingston, Ontario, Canada, for storage prior to analyses.

Preparation of samples for ^210^Pb gamma dating followed Schelske et al. (1994). To summarize, ~20 sediment intervals were strategically selected throughout each core, freeze-dried, placed into vials to a height of about 2.5 cm, and sealed with epoxy resin. Samples were allowed to rest for at least 14 days to achieve secular equilibrium between ^226^Ra and ^214^Bi. An Ortec® high purity Germanium gamma spectrometer was used to measure the activities of radioisotopes ^210^Pb, ^137^Cs, and ^214^Bi (proxy for supported ^210^Pb) at PEARL. The CRS model (Appleby 2001) was used to establish core chronology. For the Nipissing 1 core, stable lead concentrations were measured by ICP-OES (inductively coupled plasma—optical emission spectrometry) by the Analytical Services Unit at Queen’s University, and profile features (i.e., the rise and peak concentrations) were used as independent dating markers (for ~1890 and ~1970, respectively; Blais et al. 1995). Water content of intervals was estimated from weight loss following freeze-drying of the ~20 samples. Organic matter was not determined.

Sediment was heated in a 5% KOH solution for 20 min at about 80°C to deflocculate the samples. The samples were then rinsed through a 95-μm mesh sieve with distilled water. *B. cederströmii* remains, including mandibles, complete tail spines, and fragments of broken tail spines (Fig. 2) were hand-picked from the sediment concentrate using a Bogorov counting tray (Gannon 1971) under a dissecting microscope. Each tray was searched four times. The first two passes focused on the top and bottom of the channel, respectively. The contents were then mixed and the same two passes were repeated. Remains were plated and mounted onto microscope slides using Entellan® as a binding agent and examined using a Leica DMR HC light microscope at 200–400× magnification. All glassware, the searching tray, sieve, and forceps were thoroughly washed between samples and visually inspected (under a microscope when required) to ensure that no cross-contamination occurred between intervals.

For Three Mile Lake we also searched sediments from a core collected in 1994, prior to the year of first detection of *B. cederströmii* in plankton net tows in 2001. The methods of collection for this core are the same as those reported above for the other Ontario cores and the collection location is reported in Table 1 under Three Mile Lake H1. The historical core material was stored dry and was processed and searched for *B. cederströmii* remains as described above for the other Ontario cores.

### Temperature data

Mean July air temperature data were obtained for the region surrounding each lake to assess if a possible temperature anomaly was synchronous with changes in accumulation rates of *B. cederströmii* remains in the sediment (Walsh et al. 2016b). Air temperature was used as a proxy for water temperature, as water temperature data for the study lakes were not available. Air temperature data for Minnesota lakes were retrieved from National Weather Service reporting stations 214026 in International Falls, MN, near Lake Kabetogama, and 214103, twelve miles north of Isle, MN, near Lake Mille Lacs (National Weather Service 2019). Air temperature data for Ontario lakes were retrieved from the Adjusted and Homogenized Canadian Climate Data for station 6110607 Beatrice (Three Mile Lake) and 6085701 North Bay (Lake Nipissing) (Vincent et al. 2012). We restricted the time frame for historic temperature data to 1960–2019, because that was the period of consistent data reporting among the four stations.

## Results

**Fig. 3:**
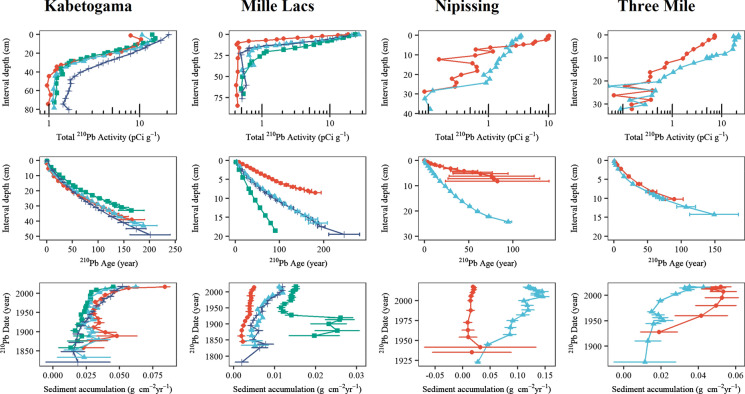
^210^Pb activity, ^210^Pb-estimated sediment age, and sediment accumulation rate for the 12 cores. The symbols for the core sites are site 1 (red circle), site 2 (blue diamond), site 3 (green square), and site 4 (black cross). Error bars represent ± one standard deviation

Recent sediments of Lakes Kabetogama and Mille Lacs have high water (89–93%) and organic (22–45% dry mass) content, typical of inland waters of the Great Lakes region (Table 1). Both water content and organic content were lower, on average, in the top 25 cm of Lake Kabetogama cores compared to Lake Mille Lacs cores. Depth profiles of unsupported ^210^Pb decline near-exponentially down core, indicating near-constant sediment accumulation and ideal conditions for modeled chronologies (Fig. 3). However, the slight inflection at the top of three Kabetogama cores suggests some surface mixing (see results for ^7^Be). Similarly, profiles in the top 2 cm of Nipissing 2 and both Three Mile scores are slightly flattened (Fig. 3). Dating uncertainty is generally low in the Minnesota cores (± 2–5 years for the twentieth century) with the oldest attainable dates extending back at least to the mid-1800s. Dating uncertainty ranges higher in the Ontario cores (up to ± 14 years), with the oldest dates extrapolated back to the 1100s with dry mass and linear sediment accumulation rates. Age-at-depth profiles range widely among the sediment cores within and between lakes (Table 1, Fig. 3), which is to be expected, as sediments commonly accumulate at different rates across the bottom of a lake as a result of variations in sediment input and focusing.

^7^Be was detected in sediment core intervals down to 2 cm in Lake Kabetogama and 3 cm in Lake Mille Lacs. Given the high sediment accumulation rates in the Kabetogama cores, this amount of mixing would have a minimal effect (< 2 years) on dates derived by the CRS model (Appleby 2001). On the other hand, in the slowly accumulating Mille Lacs cores, dates below the mixed zone could be as much as 20 years too young if the upper 3 cm of core were totally homogenized. This is unlikely the case, however, as the ^210^Pb activities are not homogenous in the upper 3 cm of the Mille Lacs profile, but rather decline steadily with depth. More critically, even partial sediment mixing could displace exoskeletal remains into strata older than the actual subfossils. Using our ^210^Pb determined sediment ages (Fig. 3), ^7^Be results indicate that exoskeletal remains could be mixed to depths of about 4 years older than the remains themselves in Lake Kabetogama and 20 years older in Lake Mille Lacs.

**Fig. 4:**
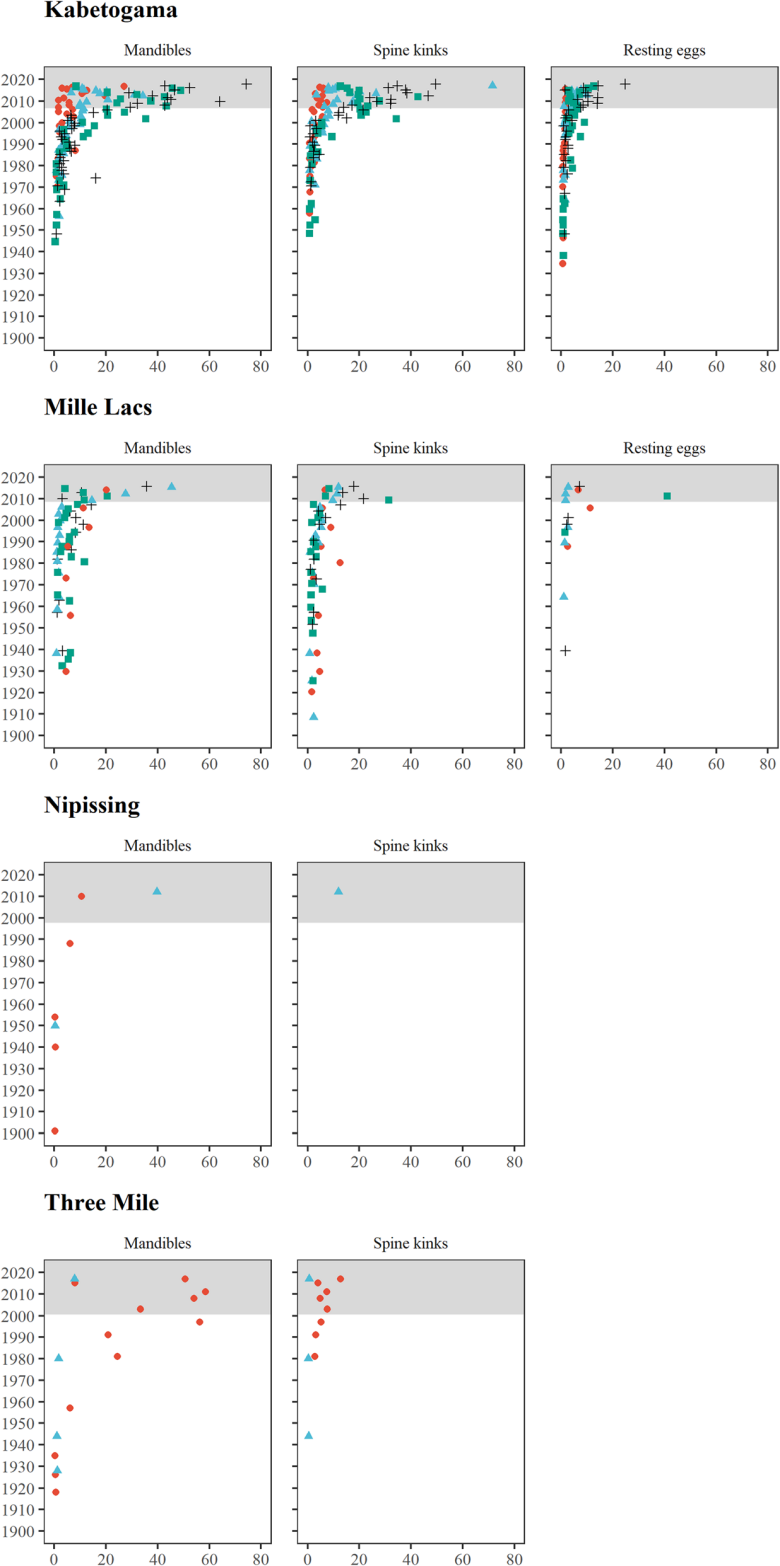
Mandibles, spine kinks, and resting eggs of *B. cederströmii* recovered from lake sediments and reported as density (number per gram sediment dry weight) as a function of estimated sediment age (determined by ^210^Pb). The symbols for the core sites are site 1 (red circle), site 2 (blue diamond), site 3 (green square), and site 4 (black cross). Gray shaded portion represents the reported invasion period based on net-tow collections. Only non-zero density values are reported. Coring location coordinates and water column depths are given in Table 1

In Lake Kabetogama and Lake Mille Lacs, Minnesota (USA), exoskeletal remains at four widely separated coring sites in each lake are continuously present from the most recent year interval (2017 or 2018) to intervals dated to the 1950s (Fig. 4). In the pre-1950s sediment, exoskeletal remains of mandibles, tail spine kinks, and resting eggs are still present, but sporadic, back to ca. 1934 (Kabetogama) and ca. 1908 (Mille Lacs). Assorted smaller exoskeletal fragments of tail spines (not shown in Fig. 4) were found throughout core intervals as far back as 1900, which are the earliest core intervals that we searched from these two lakes. Exoskeleton fragments recovered from older sediments were not noticeably thin or poorly preserved, indicating that there was no exoskeleton degradation occurring in the sediments. There were generally higher densities of mandibles and tail spine kinks than of resting eggs. This is expected if exoskeletal remains experience equal rates of settling, decomposition, and loss over time because resting eggs are generally produced only in the fall and represent a smaller fraction of the annual inventory of individuals in a population (Branstrator 2005).

In Lake Nipissing and Three Mile Lake, Ontario (Canada), we limited our searches to mandibles and tail spines, but extended our analyses to core intervals older than 1900. In Lake Nipissing, mandibles were present throughout the core intervals to as early as 1900 (Fig. 4). In Three Mile Lake, by contrast, mandibles were present not only throughout the 1900s, but multiple remains were also recovered from older sediment intervals (not shown) that date to before the 1650s (25 cm sediment depth), representing the earliest evidence recovered among the four lakes.

Among the cores from all four lakes, there were generally higher densities of mandibles than tail spine kinks. This is to be expected because individuals contain two mandibles and one tail spine, and the exoskeleton on the mandibles is shed during molting, unlike the tail spine, which is not lost during molting.

Our searches of sediments from the two historical Lake Kabetogama cores and one Three Mile Lake core (Table 1), which were collected before the first in-water detections, yielded no *B. cederströmii* exoskeletal remains. In Kabetogama H1 (collected in 2001), we searched 9.5 g dry sediment, distributed fairly evenly from 0 to 13 cm depth. In Kabetogama H2 (collected in 2005), we searched 0.7 g dry sediment, distributed fairly evenly from 1 to 6 cm depth. In Three Mile H1 (collected in 1994), we searched approximately 27.5 g dry sediment, distributed fairly evenly from 0 to 8 cm depth, with a larger amount of sediment searched from 8 to 18 cm depth. Exoskeletal remains of other cladoceran taxa, including bosminiids and daphniids, were abundant and easily recognizable in all historical cores.

**Fig. 5:**
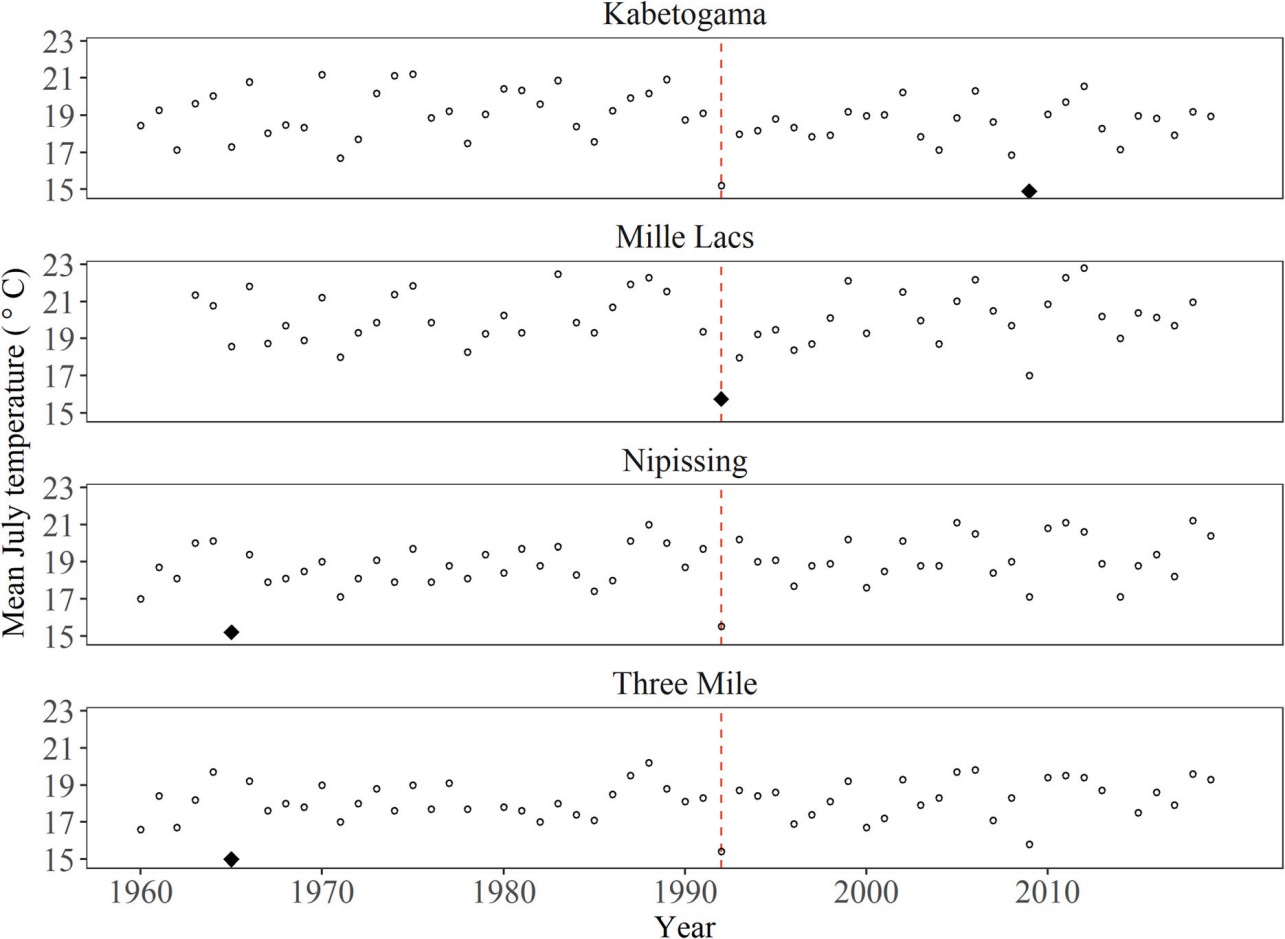
Mean July air temperature for weather reporting stations near Lake Kabetogama, Lake Mille Lacs, Lake Nipissing, and Three Mile Lake from 1960 to 2019. The coldest July for the time period is represented by a black diamond. The year 1992 is marked by a red dashed line

Densities of *B. cederströmii* exoskeletal remains began to increase around 1990–2000 in all study lakes (Fig. 4). Unseasonably cold mean July air temperatures in both Minnesota and Ontario occurred in the early 1990s (Fig. 5). There were also cold mean July air temperatures in the mid-1960s (Ontario) and in 2009 (Minnesota and Ontario).

## Discussion

Our results potentially extend evidence for the beginning of North American occupation by *B. cederströmii* from the 1980s to the early part of the twentieth century in four lakes, and much earlier in one of those lakes (Three Mile Lake, Ontario). They raise significant questions about the origin and subsequent range expansion of *B. cederströmii* on the continent and demonstrate the presence of protracted periods of time without detection by traditional plankton net tows or any other means (e.g., ensnared on angling gear). If our results are valid, they demand rethinking about the invasion dynamics of *B. cederströmii* and our methods of study. If our results are artifactual, they imply that the paleolimnological approaches that we used, otherwise widely adopted for studies of many species of crustacean zooplankton where large numbers of specimens are identified, are potentially unsuitable for the study of species invasion (such as by *B. cederströmii*), for which only presence and absence data are relied upon. We acknowledge that in typical paleolimnological investigations, the finding of one or two specimens would never be considered significant; however, when investigating a potential invader, such data (if reliable) are clearly important.

The prevailing view regarding continental invasion of *B. cederströmii* is that it became established in North America during the late 1970s to early 1980s when propagules arrived in the ballast water of ocean-going cargo ships (Sprules et al. 1990). This scenario, however, has never been confirmed with direct evidence. The purported timeframe is based on analyses of plankton-net assemblages and fish-stomach contents collected during the 1980s from the Great Lakes, which provided the first evidence of the species on the continent (Johannsson et al. 1991; Bur et al. 1986). The inferred mechanism of transport in ship ballast water is based on the timing of the opening of the St. Lawrence Seaway in 1959, which accelerated transport of ballast water from foreign cargo ships into the Great Lakes (Mills et al. 1993; Ricciardi 2006; Sturtevant et al. 2019). But shipping between the Great Lakes and the Atlantic Ocean was already underway long before 1959 and therefore it is possible that ballast water containing *B. cederströmii* could have been released into the Great Lakes (Mills et al. 1993) up to a about century earlier, following completion of the first Welland Canal around Niagara Falls (1829) and the first St. Lawrence River canal system (1847) (LesStrang 1981). By one estimate, ballast water is credited with the arrival of 6 non-native species in the Great Lakes before 1956 (Ricciardi 2006). This nuanced history of Great Lakes shipping, together with our sediment evidence, suggests that the origins of *B. cederströmii* in North America could have occurred through ballast water discharge, but at a much earlier initial date than currently described in the literature.

If our results are valid, they raise questions regarding the timing and sequence of lake invasions during continental range expansion. If *B. cederströmii* first arrived on the continent in ship ballast water, it likely first established population centers in the Great Lakes and then subsequently spread inland, possibly associated with recreational boating activities (MacIsaac et al. 2004). This scenario predicts that Great Lakes sediments should house chronological records that establish their position as a continental beachhead. However, uncovering this type of evidence seems unlikely for two reasons. First, a published study on the paleolimnology of *B. cederströmii* from Lake Erie reports that the oldest exoskeletal remains there date to near 1980 (Keilty 1988), which is inconsistent with an invasion that predates our inland lake records. Second, the lack of evidence of *B. cederströmii* among Great Lakes plankton-net surveys done throughout the 1900s argues against its presence in the Great Lakes prior to inland lakes (Wells 1970; Watson and Wilson 1978). An alternative scenario of continental range expansion is that *B. cederströmii* initially arrived by a vector other than ship ballast water and initially established population centers in inland lakes, only later spreading to the Great Lakes.

Although still tentative, our single record that places *B. cederströmii* in North America pre-1650s (Three Mile Lake, Ontario) hints at the possibility that the genus could be native. However, this is a speculative hypothesis. Distinguishing between the two aforementioned scenarios of continental range expansion will require more evidence, some of which might be gathered through paleolimnological work on millennial time scales from many lakes and locations. Notably, these scenarios of range expansion are not mutually exclusive, making it possible that *B. cederströmii* in North America is an ensemble of multiple origins and gene pools, some of which may have arrived recently from Eurasia (Colautti et al. 2005). Although extant populations of *Bythotrephes* in North America represent the morphological species *B. cederströmii* (Korovchinsky and Arnott 2019), not all populations have been studied, including historical ones, leaving open the possibility that multiple morphological species have existed through time, or may currently coexist on the continent.

Our results also indicate that *B. cederströmii* populations persisted in lag phases (decades of prolonged low abundances) before escalating in the 1990s and 2000s (Fig. 4). Lag phases are characteristic of invasive species, though their causes (e.g., poor environmental conditions, and genetic and reproductive barriers) can be elusive (Simberloff 2009). In the case of *B. cederströmii*, climate anomalies, reductions in predation-based mortality, genetic subsidy from Eurasia, or reduction in mating barriers could all have possibly triggered their population expansions (Walsh et al. 2016b; Manca and DeMott 2009; Wittmann et al. 2011). Commonality in the time period (1990s and 2000s) when *B. cederströmii* transitioned from low to high abundance in the four inland lakes points to the likelihood of a single, regional triggering mechanism. A cooler than average July mean temperature is one possibility that is supported by circumstantial evidence. Data show that the entire region experienced low temperatures in the summer of 1992, around the time that accumulation rates of exoskeletal remains began to escalate across the study lakes. This is around the year that *B. cederströmii* also began appearing in the water column for the first time in inland lakes in Canada and the USA (Yan et al. 1992; Branstrator et al. 2006). Whether the close timing of cool temperatures and first appearances is causal or coincidental remains unclear. Walsh et al. (2016b) pointed to a cool summer in 2009 as the triggering mechanism that led to a surge in *B. cederströmii* density and its first detection in the water column of Lake Mendota, Wisconsin (USA). The mechanism by which cool temperatures drive a release of *B. cederströmii* from a lag phase may involve a thermal preference (Grigorovich et al. 1998), a dissolved oxygen preference (Sorensen and Branstrator 2017), or changes in predatory regime (Walsh et al. 2016b). Some fish species have declined in abundance in some of our study lakes over the last several decades (Anthony and Jorgensen 1977; Venturelli et al. 2014). However, the correlation between predatory regime changes through a decline in fish abundance and an increase in *B. cederströmii* abundance needs further study. In contrast to our results, two previous studies that used methods similar to ours report < 16 years between first presence of *B. cederströmii* in the sediments and first detections in the water column (Walsh et al. 2016b; Branstrator et al. 2017), which underscores the fact that long lag phases are not required for establishment of *B. cederströmii* populations.

Shortly after *B. cederströmii* was first recorded in North America, paleolimnology was heralded as a method that could be used to document the timing of lake invasions (Keilty 1988). The species’ barbed tail spine, which is diagnostic and preserves well in lake sediments, gave promise to the prospect that its arrival and presence in a lake could be reconstructed through analysis of exoskeletal remains. To date, however, only four published studies in North America have used paleolimnology to assess *B. cederströmii* invasions (Keilty 1988; Hall and Yan 1997; Walsh et al. 2016b; Branstrator et al. 2017). Therefore, we are still learning whether the approach of identifying very low numbers of specimens has vulnerabilities and incongruities with other methods when used to reconstruct the historical presence. In particular, we remain challenged to explain how *B. cederströmii* could have persisted for so long in these lakes without detection by plankton nets or anecdotal observation. Populations of *B. cederströmii* in invaded lakes vary in abundance during the growing season, but were continuously found in the water column in two studies examining seasonal abundance of the species (Brown et al. 2012; Yan et al. 2001). In Lake Kabetogama, extensive zooplankton monitoring with plankton nets from 1978 to 2003 failed to record a single *B. cederströmii* specimen (Kallemeyn et al. 2003; Kerfoot et al. 2016). However, given our sediment-based reconstructed estimates of the densities of *B. cederströmii* living in Lake Kabetogama during 1978–2003 (DeWeese 2020), such a failure of detection by plankton nets during this time period is statistically unlikely (Walsh et al. 2018). Likewise, in Lake Mille Lacs, aggressive zooplankton monitoring by net tows that began in 2006 by the Minnesota Department of Natural Resources staff failed to detect *B. cederströmii* until 2009 (DeWeese 2020). Moreover, both Lake Kabetogama and Lake Mille Lacs are common destinations for sport anglers, and the lack of any anecdotal evidence of an earlier presence of *B. cederströmii*, despite statewide awareness campaigns since the early 2000s, is puzzling and points to a gap in our understanding of the relationship between the neo- and paleo-records.

In Eurasia, where *Bythotrephes* is native, researchers have documented its exoskeletal remains and resting eggs in numerous lakes and to great (> 50 cm) sediment depths (Herzig 1985; Nilssen and Sandoy 1990; Milan et al. 2017). However, even there, exceptions occur. For example, in Lago Maggiore, Italy, *Bythotrephes* are easily detected with plankton nets, but two independent paleolimnological studies failed to find a single piece of exoskeletal evidence in sediment cores from the lake (Manca et al. 2007; Nevalainen et al. 2018). Possible explanations for the absence of sedimentary evidence of *Bythotrephes* in Lago Maggiore include poor exoskeletal preservation and spatial patchiness. *Bythotrephes* has a low calcium content compared to other cladocerans (Kim et al. 2011), which may explain differences in its sedimentary preservation. Additionally, previous studies have found that other cladoceran exoskeleton preservation may vary throughout the sediment record due to physical and biological factors, including fish consumption (Leppänen and Weckström 2016). This gap between the neo- and paleo-records in Lago Maggiore, like our results, is cautionary and points to the presence of still unknown relationships between living *Bythotrephes* populations and the fate of their sedimentary remains.

If the early timelines of *B. cederströmii* presence suggested by our results are artifactual, it is incumbent on us to identify the sources of error and consider how these could be resolved in future studies. In the interpretation of paleo-records of other crustacean zooplankton, it has been noted that sediment focusing and resuspension, as well as exoskeletal preservation, can vary by species and inter-annually (Korhola and Rautio 2001; Nykänen et al. 2009). For our purposes, we grouped the possible sources of error as: (1) those associated with the procedures and equipment that we used for core collection, processing, and analysis; and (2) those associated with natural redistribution of exoskeletal remains in lake sediments prior to our core collections.

In order to minimize error associated with core collection, processing, and analysis, our two teams used procedures and equipment that have been widely vetted in the discipline of paleolimnology. One procedural distinction of note between our two approaches was that during the extrusion step of core processing we trimmed and discarded the outer 0.5-cm edge of the intervals of the sediment cores collected from Lake Kabetogama and Lake Mille Lacs (USA), but not those collected from Lake Nipissing and Three Mile Lake (Canada). Trimming should have eliminated any material translocated along the core barrel walls during collection and extrusion.

If, however, smearing did extend inward farther than 0.5 cm it could have affected our timelines of species presence. Only further study can resolve whether this is of concern. If smearing leads to a predictable rate of decline in numbers of fragments downcore, this could possibly be used as a null hypothesis against which core data could be compared. The great length (> 0.5 cm) and barbed anatomy of the tail spine of *Bythotrephes* might increase its likelihood of ensnaring on surfaces and smearing, and this could present special problems for paleolimnological reconstruction. However, our extensive taxonomic evidence for *B. cederströmii* presence also includes small fragments of the tail spine, as well as mandibles and resting eggs (Fig. 2), none of which is unique in their size or anatomy compared to those of other crustaceans commonly studied using sediment cores. Regardless of the remains considered, the evidence suggests early presence in each of the four study lakes. It could be hypothesized that we incorrectly identified the exoskeletal remains of *B. cederströmii*, but that is highly unlikely given the unique morphological characteristics of the tail spine and mandible. Nonetheless, to be conservative we reported only the most diagnostic forms of the remains (mandibles, tail spine kinks, and resting eggs), and not the large numbers of additional tail spine fragments in the samples that lacked the kinked portion.

In addition to possible translocation of exoskeletal remains by core-wall smearing, the physicality of core collection and transport to the laboratory for the Minnesota cores (the Ontario cores were extruded on shore) could have redistributed exoskeletal remains down core. To minimize this, we applied Zorbitrol (Tomkins et al. 2008) to stabilize the sediment–water interfaces of the Minnesota cores immediately post-collection. Those cores were transported to the laboratory and stored horizontally in a walk-in refrigerator before analysis. Occasional visual inspection of the cores did not reveal any movement in the sediment or pore water along the core walls. Possible movement of exoskeletal remains could have happened in the interior regions of the sediment matrix that we could not ascertain by visual inspection. Whereas some minor movement of exoskeletal remains by this mechanism cannot be ruled out, the core profiles of ^210^Pb and ^7^Be are inconsistent with substantial sediment mixing. In addition, major changes in the microfossil assemblage of diatoms and midges that were evident mid-core in Lake Nipissing site 1 (Favot 2021) suggest that any mixing by coring, transporting, or extruding was not pronounced.

Our ^210^Pb dating results indicated that sedimentation rates in the top 25 cm of the cores differed by a factor of about 2 to 3 between Lake Kabetogama and Lake Mille Lacs (Fig. 3). Consequently, the depths of the time horizons dating to equivalent ages (e.g., the year 2000, 1980, or 1930; see Table 1) ranged accordingly. If *B. cederströmii* did first invade Lake Kabetogama in 2007 and Lake Mille Lacs in 2009 (which are dates of first water column records, respectively), it follows that the entirety of the inventories of exoskeletal remains that we recovered from earlier sediments in both lakes are artifactual. If so, it is curious that in both lakes we see a surge in densities of exoskeletal remains around 1990–2000. For this to be the case, significant numbers of exoskeletal remains would have had to translocate about 2–3 times deeper in the sediments of Lake Kabetogama than Lake Mille Lacs. This demands extraordinary and consistent errors that seem untenable. Moreover, the water and organic contents in the top 25 cm of Lake Kabetogama sediments were less than those in Lake Mille Lacs sediments (Table 1) and this should have reduced their vulnerability to mixing. Dating error, another possible artifact, can largely be ruled out as an explanation for the early appearance of exoskeletal remains in our cores. The exponential ^210^Pb activity profiles and low uncertainty associated with twentieth century dates (< 5 years) precludes timing errors of that magnitude.

As a second potential source of error, we considered whether physical resuspension at the sediment–water interface and bioturbation could have redistributed *B. cederströmii* exoskeletal remains prior to our core collections. A wide taxonomic range of macroinvertebrates, and some fish, disrupt sediments through occupancy (e.g., burrowing) and feeding, which can redistribute constituent particles, including exoskeletal remains of crustacean zooplankton (Adámek and Maršálek 2013). *Chaoborus* commonly burrow up to 3 cm depth (LaRow 1969; Gosselin and Hare 2003), and *Hexagenia*, which is among the deepest burrowers, may excavate tunnels up to 10 cm depth in lake sediments (Charbonneau et al. 1997). In three of our four lakes (Mille Lacs, Nipissing, and Three Mile), these burrowing depths could be sufficient to account for redistribution of exoskeletal remains to at least the 1980 horizons, but in Lake Kabetogama the 1980 horizon is too deep for disruption by such burrowing activity (Table 1).

To further explore whether natural sediment mixing (e.g., by wave action, porewater advection, and bioturbation) could help explain the patterns in exoskeletal remains, we analyzed ^7^Be decay profiles to estimate depths of modern, near-surface mixing in Lake Kabetogama and Lake Mille Lacs. Our results indicate that modern sediment mixing is confined to the top 2 cm in Lake Kabetogama and top 3 cm in Lake Mille Lacs. Given our sediment age-at-depth calculations for Lake Mille Lacs, the results imply that exoskeletal remains there could have been mixed to depths at which the sediment is nearly 20 years older than the age of the remains themselves. In Lake Kabetogama, however, where sedimentation rates are faster, exoskeletal remains could have been mixed by natural processes to depths at which sediments are only 4 years older, at most, than the age of the remains themselves. Even if we postulate that *B. cederströmii* were first present, died, and settled to the lake sediments in the 1980s (the decade of first detection of *B. cederströmii* on the North American continent; Johannsson et al. 1991), our ^7^Be-based estimates of modern sediment mixing in each lake, extrapolated across a respective core’s history, cannot explain the stratigraphic patterns. The only exception to this is with core site 1 from Lake Mille Lacs where low sedimentation rates yielded a sediment age of 1930 at 5 cm depth, which is only 3 cm below the 1980 horizon (Table 1). Although Hall and Yan (1997) demonstrated that *B. cederströmii* tail spines do not move vertically in sediments in Harp Lake, Ontario, their study was conducted within 1–2 years of first records of *B. cederströmii* in the lake, and it is possible that longer residency could cause deeper redistribution of remains if the phenomenon is time-dependent.

We also examined historical cores collected prior to *B. cederströmii* detection in open water to supplement our findings from recently collected cores. We expected to find exoskeletal remains in historical cores at similar ages and densities as the recently collected cores; however, we failed to recover any *B. cederströmii* remains. The complete absence of *B. cederströmii* exoskeletal remains suggests that sediment mixing may have occurred, although we took thorough steps to prevent mixing during core collection and extrusion and our isotope analysis did not indicate substantial mixing had occurred. These results remain a confounding element in our analysis.

In conclusion, ecological studies have long been plagued by inadequate methods to detect species that are small-bodied, behaviorally cryptic, low in abundance, or limited in distribution (Hoffman et al. 2011; Walsh et al. 2017). This gap has impeded our ability to reconstruct pathways and timelines of range expansion, and adequately document the distribution of biodiversity, for native and non-native species alike. In view of this, our results, if valid, highlight the potential that paleolimnology holds for the discovery and assessment of rare species in aquatic environments. The four lakes that we studied are popular tourist and angling destinations, and some are well-studied scientifically. Despite considerable management attention, *B. cederströmii* was not recorded in them until the 1990–2000s. If our results are valid, they suggest that sediment records may be far more sensitive to small founder populations than traditional detection tools (Walsh et al. 2017). Such prolonged lags in the discovery of invasive species have broad implications for the capacity of humans to manage this dimension of global environmental change (Vitousek et al. 1997).

Nonetheless, our results raise a number of issues that will eventually demand reconciliation. These include: (1) the lack of a clear narrative of continental invasion that is congruent with ballast water shipping and the timing of first records in the Great Lakes and inland lakes, (2) the lack of a clear mechanism for a transition from a lag phase to a growth phase in our four lakes, and (3) the lack of evidence of *B. cederströmii* using traditional methods prior to the 1980s in North America. For these reasons, we suggest that the early timelines for presence suggested by our results may be artifactual, and therefore should be regarded as ‘preliminary findings’ at this time.

We should emphasize that, in any typical paleolimnological assessment, the simple presence of one or two specimens would never be considered definitive, except for the special case of tracking an initial invasion (as we have attempted here). If indeed our seemingly early detection of *B. cederströmii* in all four of our lakes is an artifact, it does not call into question the large number of paleolimnological studies that have successfully used these approaches to reconstruct changes in zooplankton community structure, since most studies exclude very small numbers of remains. Future studies could help validate our results. Experiments that determine whether exoskeletal remains of *B. cederströmii* preferentially translocate downwards in sediments in response to biological and physical disruption, including during core collection and/or extrusion, could reveal errors in our methods and the nature of how lake sediments archive *B. cederströmii* remains. Additionally, examination of *B. cederströmii* in historical plankton collections, fish stomachs (e.g., museum-archived specimens), or sediment cores that were collected prior to dates of first records in the water column could help support or refute our results, which point to early presence. Until further study, however, we caution that paleolimnology may not be a secure method to pinpoint very early detection and assess initial timelines of colonization of *B. cederströmii* in ecosystems, if numbers are extremely low as they were in our cores.
